# Erythropoietin overrides the triggering effect of DNA platination products in a mouse model of Cisplatin-induced neuropathy

**DOI:** 10.1186/1471-2202-10-77

**Published:** 2009-07-15

**Authors:** Min-Suk Yoon, Zaza Katsarava, Mark Obermann, Maria Schäfers, Bernd Liedert, Anna Dzagnidze, Andreas Kribben, Rupert Egensperger, Volker Limmroth, Hans-Christoph Diener, Juergen Thomale

**Affiliations:** 1 Department of Neurology, University of Duisburg-Essen, Hufelandstr. 55, 45122 Essen, Germany; 2 Department of Nephrology, University of Duisburg-Essen, Hufelandstr. 55, 45122 Essen, Germany; 3 Institute of Pathology and Neuropathology, University of Duisburg-Essen, Hufelandstr. 55, 45122 Essen, Germany; 4 Institute for Cell Biology, University of Duisburg-Essen, Hufelandstr. 55, 45122 Essen, Germany

## Abstract

**Background:**

Cisplatin mediates its antineoplastic activity by formation of distinct DNA intrastrand cross links. The clinical efficacy and desirable dose escalations of cisplatin are restricted by the accumulation of DNA lesions in dorsal root ganglion (DRG) cells leading to sensory polyneuropathy (PNP). We investigated in a mouse model by which mechanism recombinant erythropoietin (rhEPO) protects the peripheral nervous system from structural and functional damage caused by cisplatin treatment with special emphasis on DNA damage burden.

**Results:**

A cumulative dose of 16 mg cisplatin/kg resulted in clear electrophysiological signs of neuropathy, which were significantly attenuated by concomitant erythropoietin (cisplatin 32,48 m/s ± 1,68 m/s; cisplatin + rhEPO 49,66 m/s ± 1,26 m/s; control 55,01 m/s ± 1,88 m/s; p < 0,001). The co-application of rhEPO, however, did not alter the level of unrepaired cisplatin-DNA lesions accumulating in DRG target cells. Micro-morphological analyses of the sciatic nerve from cisplatin-exposed mice showed damaged myelin sheaths and mitochondria. Co-administered rhEPO inhibited myelin sheaths from structural injuries and resulted in an increased number of intact mitochondria.

**Conclusion:**

The protective effect of recombinant erythropoietin is not mediated by reducing the burden of DNA platination in the target cells, but it is likely to be due to a higher resistance of the target cells to the adverse effect of DNA damage. The increased frequency of intact mitochondria might also contribute to this protective role.

## Background

The antineoplastic efficacy of treatment regimens with cisplatin (cis-diaminodichloro-platinum [II]) is hampered by its severe neurotoxic side-effects [1], which frequently limit the continuation of treatment cycles. Patients complain of distal paresthesis, loss of joint position resulting in sensory ataxia [2,3]. Sural nerve biopsies showed degeneration of large myelinated axons with signs of segmental demyelinization and remyelinization [3,4]. Electrophysiological findings revealed decreased sensory nerve conduction velocity and SAP-amplitudes [5,6].

The anti-tumor activity as well as the toxicity in physiological cells are mediated by the formation of specific cisplatin-DNA adducts with guanine-guanine intrastrand cross-links (cisPt(NH_3_)_2_d(pGpG; Pt-GG), representing the majority (~70%) of the DNA platination products. Accumulation and persistence of these DNA lesions can interfere with replication or transcription and trigger apoptotic processes [7,8]. To counteract the deleterious effects of DNA damage, mammalian cells are equipped with various DNA repair mechanisms. The most efficient pathway to remove Pt-GG adducts is nucleotide excision repair (NER) [9,10].

In a mouse model of cisplatin-induced neurotoxicity we have recently shown that Pt-GG adducts are not completely eliminated from the nuclear DNA of distinct neuronal cell types [11]. Repeated application of cisplatin resulted in an accumulation of persisting DNA cross-links in DRG neurons in parallel to decreased sensory nerve conduction velocities and their corresponding amplitudes. These effects were significantly accelerated in mice with a functional defect in NER [11].

Neuroprotective strategies might aim either at a reduced accumulation of drug-induced DNA adducts or at improved survival or function of critical cells despite of their actual damage burden. There is increasing evidence for the protective properties of erythropoietin (EPO) in neuronal tissues [12-16]. Such effects have been demonstrated both for intrinsic EPO as well as for pharmacological application of rhEPO [17,18]. Erythropoietin is a pleiotropic cytokine mediating its hematopoietic effects via homodimeric erythropoietin receptors (EpoR), which are expressed also in various neuronal tissues [13,19-21]. In the peripheral nervous system EPO and EpoR were proven to be present in DRG neurons, axons and Schwann cells [13]. Peripheral nerve injury led to augmented levels of both molecules [13,21] and to the activation of an "axono-protective" pathway via EpoR ligation in neurons [21].

By employing behavioral, electrophysiological and micro morphological techniques, we have investigated in our mouse model the potency of rhEPO to inhibit the peripheral neuropathy during exposure to cisplatin. To further elucidate the underlying mechanism, we have analyzed, in particular, whether co-application of this cytokine interfered with the accumulation of Cisplatin-DNA adducts in DRG neurons.

## Results

### Recombinant human erythropoietin extenuates cisplatin-induced neuropathy

**Figure 1:**
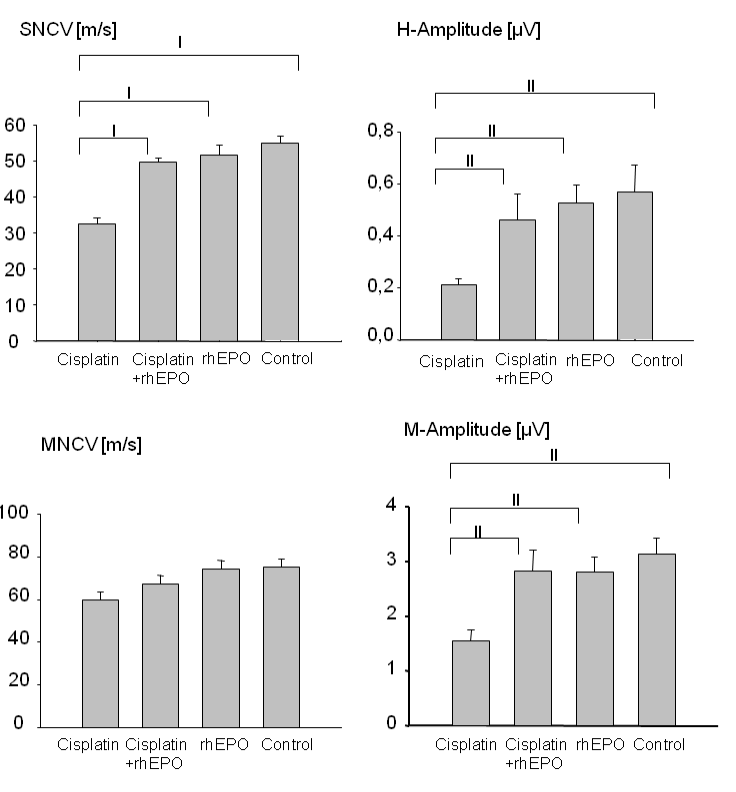
**Impact of recombinant erythropoietin on cisplatin-induced neurophysiological impairment**. C57Bl/6 mice were repeatedly injected with cisplatin (2 mg/kg, i.p; 1×/week) or with cisplatin plus rhEPO (40 μg/kg, s.c.; 3×/week) or with rhEPO alone (40 μg/kg, sc; 3×/week) for 8 weeks. The control group was injected with saline alone (500 μl i.p.; 1×/week). Electrophysiological examinations of sensory nerve conduction velocity (SNCV) and motor nerve conduction velocity (MNCV) and the corresponding M- and H-amplitude were performed in week 9. Columns represent group (n = 10) mean values ± SEM. Differences were highly significant (p < 0.001; multiple t-test with Bonferroni corrections) for SNCV values of cisplatin vs. control, for cisplatin vs. cisplatin + rhEPO groups and for cisplatin vs. rhEPO and were marginally affected for MNCV values of cisplatin vs. control groups (p > 0.05). Differences between the corresponding M- or H-amplitudes of cisplatin vs. control, for cisplatin vs. cisplatin + rhEPO and cisplatin vs. rhEPO groups were significant as well (p < 0.05). All other differences for SNCV or MNCV values were not significant (p > 0.05). I: p < 0.001; II: p < 0.05.

Repetitive applications of cisplatin (2 mg/kg/week) for 8 weeks resulted in a highly significant decrease of sensory nerve conduction velocity (SNCV) by ~40% compared to control (Figure 1). In our experiment rhEPO had a partial but highly significant protective effect despite values being different from controls (cisplatin 32,48 m/s ± 1,68 m/s; cisplatin + rhEPO 49,66 m/s ± 1,26 m/s; rhEPO 51,6 m/s ± 2,81 m/s; control 55,01 m/s ± 1,88 m/s; ANOVA df = 3, F = 25; p < 0,001). The corresponding H-amplitude was decelerated by ~60% compared to controls (cisplatin 0,21 μV ± 0,02 μV; cisplatin + rhEPO 0,46 μV ± 0,1 μV; rhEPO 0,53 μV ± 0,07 μV; control 0,57 μV ± 0,1 μV; ANOVA df = 3; F = 4,4; p < 0,05). In accordance with earlier findings [11] the nerve conduction velocities for the motor function (MNCV) were only slightly decelerated after a cumulated dose of 16 mg cisplatin/kg, however the corresponding M-amplitude was again significantly reduced compared to controls (cisplatin 1,55 μV ± 0,2 μV; cisplatin + rhEPO 2,82 μV ± 0,39 μV; rhEPO 2,81 μV ± 0,27 μV; control 3,14 μV ± 0,29 μV; ANOVA do = 3; F = 6,3; p < 0,05).

### No renal damage following cisplatin treatment

As nephrotoxicity is a common side effect of cisplatin chemotherapy all mice were given i.p. injections of 0.5 ml saline after each single dose of cisplatin. To further exclude that renal failure might modulate the nerve conductivity we determined parameters for kidney function in animals from all groups. Significant differences were observed neither for the serum creatinine status nor for the creatinine clearance (data not shown). This finding was confirmed by histopathological examinations of the kidneys showing no signs of renal damage and a normal morphology of glomerulae, microvillus and tubulus epithelium cells in all tissue samples examined.

### Mechanical sensitivity is improved by rhEPO

In addition to decreased nerve conduction velocity and sensory loss, the peripheral neuropathy can paradoxically also be associated with allodynia as observed in patients and in animal systems [22]. To analyze whether the co-application of rhEPO also modulates the somatic hypersensitivity we measured the withdrawal threshold after mechanical stimulation of the hind paw.

**Figure 2:**
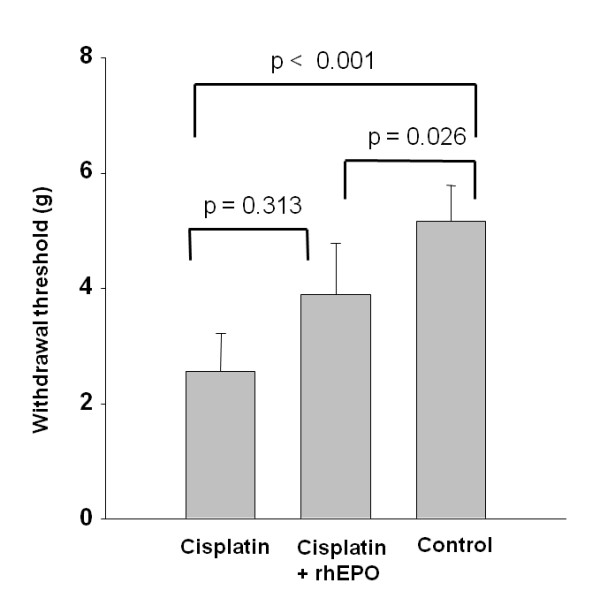
**Recombinant erythropoietin partly preserved the tactile sensitivity**. Mice were treated as in Figure 1 and were examined in week 9 for the mechanical sensitivity using the von Frey hair method and recording of the 50% withdrawal threshold. Columns represent group mean ± SEM from repeated measurements of 10 animals per group. Mechanical withdrawal thresholds were significantly reduced in the cisplatin group (p < 0.001, multiple t-test with Bonferroni corrections). The difference between cisplatin + rhEPO vs. control group was also significant (p = 0.026), while the comparison of cisplatin vs. cisplatin + rhEPO group indicated a restoration of the tactile sensitivity but lacked statistical significance (p = 0.313).

Mice treated with a cumulative dose of 16 mg/kg cisplatin showed clear signs of somatic hypersensitivity, resulting in a significant reduction in withdrawal threshold as compared to controls (Figure 2). Concomitant treatment with rhEPO lacked statistical significance (p = 0.313). Mice treated with rhEPO alone were not tested to minimize the amount of experimental animals since electrophysiological alterations were not observed.

### Accumulation of Cisplatin-DNA adducts in DRG cells

**Figure 3:**
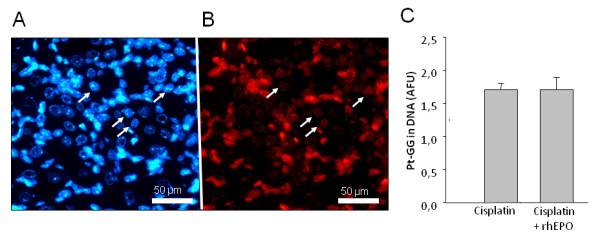
**Immunostaining of DRG and measurement of Pt-GG adduct level**. Visualization and measurement of Pt-(GG) intrastrand cross-links in the nuclear DNA of DRG neurons of C57 Bl/6 mice after chronic treatment with cisplatin (cumulative dose: 16 mg/kg). Cryosections of dorsal root ganglia were immunostained with monoclonal antibody R-C18 for Pt-(GG) adducts (B) and with DAPI for nuclear DNA (A). Arrows indicate nuclei of neurons. C: Measurement of Pt-(GG) adduct levels in DRG neurons of mice treated with Cisplatin alone or in combination with rhEPO by quantitative image analysis of nuclear signals (AFU: arbitrary fluorescence units normalised for the DNA content of individual cells); means of >200 nuclei +/- SD.

The formation of drug-induced DNA adducts is assumed to initially trigger apoptotic processes in DRG cells finally resulting in sensory neuropathy [23,24]. Employing a recently established monoclonal antibody-based immunoassay coupled with quantitative image analysis enabled us to measure structurally defined DNA platination products in individual cell nuclei [25]. The method was previously shown to provide dose-dependent results in cisplatin-exposed cells as well as in various tissue sections from treated mice. The accumulation of unrepaired Pt-GG lesions in DRG neurons during repetitive exposure to cisplatin coincided with the onset of PNP in mice [11]. Here we investigated the role of rhEPO in modulating the rates of formation or repair of DNA lesions and measured the adduct concentration in the nuclei of DRG cells (Figure 3). After a cumulative dose of 16 mg cisplatin/kg the mean values of Pt-GG levels in DRG neurons did not exhibit significant differences (1,7 ± 0,1 vs. 1,7 ± 0,2; mean ± SD) between mice treated with cisplatin alone or in combination with rhEPO (Figure 3C).

### Histopathological alterations

**Figure 4:**
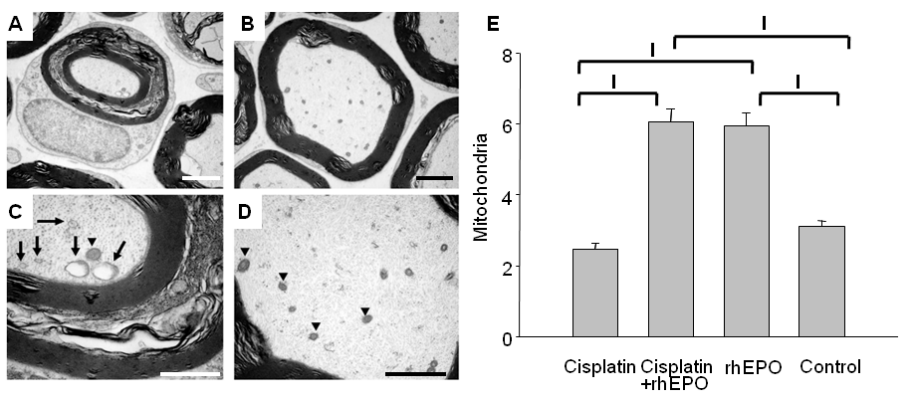
**Axonoprotection, mitochondrial protection and increased frequency of mitochondria by rhEPO**. Electron microscope scanning of the sciatic nerve from mice treated with cisplatin (A, C) or with cisplatin + rhEPO (B, D) show axonal degeneration with impaired mitochondria only in A and C. Here mitochondria show an increased volume and loss of integrity (arrows in C; arrowheads in C and D indicate intact mitochondria). Co-treatment with rhEPO resulted in significantly higher numbers of intact mitochondria within the axon (E) as compared to the cisplatin and the control group. Columns represent the mean number of mitochondria/axon section in 150 axons. To avoid redundant figures, axons with increased numbers of intact mitochondria with respect to rhEPO injection alone are not shown and to minimize the amount of figures lower magnification were excluded. Magnification in A, B: 7000-fold, C, D: 12.000-fold. Scale bar = 2 μm. I: p < 0.001.

To examine whether the functional impairment of the sensory nerve system after repetitive treatment with cisplatin is associated with morphological alterations or sub-cellular damage in the pertaining tissues, we analyzed sections of the sciatic nerve by electron microscopy. Various degenerative alterations were found in tissue sections from mice of the cisplatin group with abnormalities in myelinated fibers to be most prominent (Figure 4A). The layers of myelin sheaths were split up. Additionally, the diameter of mitochondria was increased in those axons and edema or lysis of christae was obvious (Figure 4C). In both groups treated with rhEPO the splitting of myelin sheaths was not observed (Figure 4B). Remarkably, in both groups treated with rhEPO a significant increase of intact mitochondria was observed in the axoplasm compared to cisplatin- and control-group (Figure 4E). In summary, i) morphologically damaged mitochondria were observed solely in neuronal cells of the cisplatin group and were absent in rhEPO treated group and ii) in both groups treated with rhEPO a significant increase of intact mitochondria was observed.

## Discussion and conclusion

In the current study we found that rhEPO ameliorated cisplatin-induced neuropathy in mice. As previously shown, we found no effect on electrophysiological parameters by rhEPO alone [26]. The compound improved electrophysiological and morphological parameters, but did not interfere with the accumulation of Pt-DNA adducts in critical neuronal cells. Our findings suggest that the neuroprotective effects are mediated by improving the cell resistance to side effects of Cisplatin rather than by influencing the formation or repair rates of cisplatin-induced cross-links in the nuclear DNA of DRG cells.

The primary target structures in the nervous system are the DRG cells and loss of function or cell apoptosis in DRG cells are hallmarks of sensory neuropathy [24]. Spectroscopic post mortem analysis of various neuronal tissues from patients treated with cisplatin revealed high concentrations of platinum in the DRG [3]. More recent studies confirmed this by demonstrating augmented levels of platination products in the nuclear DNA of DRG neurons and satellite cells of cisplatin-exposed mice [27]. By analyzing two syngenic mouse strains [11] either proficient or deficient for nucleotide excision repair (NER), we showed that the severity of cisplatin-induced neuropathy was significantly correlated with the amount of persisting Pt-DNA adducts in DRG cells. Our findings suggest a possible relationship between the extent of DNA platination and the degree of sensory impairment.

As the NER mechanism does not only take care of the integrity of the bulk nuclear DNA but is even more efficient in removing damage from actively transcribed genes, it is still unclear whether persisting cisplatin adducts in that sub-fraction of the genome play a particular role in triggering functional loss or demise of DRG cells. Due to the excessive energy consumption of neuronal cells and the observed structural damage in mitochondria, drug-induced damage in the mitochondrial DNA (mtDNA) has been suggested to cause the functional impairment. However, as the NER system efficiently protects mice from cisplatin-induced neuropathy by not acting on the mitochondrial genome [28], it is very unlikely that accumulated platination products in mtDNA play a major role in this process.

Until now, the direct molecular link by which persisting intrastrand cross links in nuclear DNA trigger the loss of mitochondrial integrity and the functional impairment in cisplatin-exposed neuronal cells is unclear. Experiments with explanted, cisplatin-exposed DRG neurons demonstrated the translocation of cytosolic Bax protein to the mitochondrial membrane with consecutive release of cytochrome c from this organelle and the activation of caspase 9 [29]. In this model the initial sensor for DNA platination products remains obscure as well as the putative mechanism by which EPO exerts its protective effects.

Another attractive model encompasses the mutual antagonistic regulation of apoptosis and autophagy in mammalian cells [30]. The balance between both mechanisms is determined amongst others by the minor cytosolic fraction of the High-mobility group 1 protein (HMGB1) which is a structurally conserved nuclear protein acting as an architectural chromatin binding factor. As HMGB1 has been shown to strongly bind to cisplatin-adducted nuclear DNA [31], it can be hypothesized that the level of free HMGB1 in the cytoplasm is diminished by such a "nuclear trapping" process and, thereby, autophagy is repressed, mitochondrial integrity is lost and apoptotic mechanisms are activated.

In our mouse model we have demonstrated that the sciatic nerve revealed severe structural damage of the myelin sheaths and the mitochondria in cisplatin treated mice. Likewise, in streptozotocin-induced rats retinal neuronal cells show similar morphological changes in mitochondria that are diminished by rhEPO [32]. Parallel to mitochondrial protection rhEPO saved the myelin sheaths from structural damage (Figure 4). A similar axon protective effect of rhEPO had been suggested previously [21]. Schwann cells were shown to abundantly express EPO receptors and ligand binding led to the phosphorylation of the Janus kinase-2 (JAK2) [19], which is associated with anti-apoptotic processes. In a spinal nerve crush model rhEPO protected DRG neurons against neuronal apoptosis by enhanced JAK2 phosphorylation [13]. Proteins of the bcl-2 family, extracellular signal-regulated kinases and the phosphatidylinositol 3-kinase/Akt seemed to be involved in EPO-activated anti-apoptotic pathways as well [33,16]. Binding of erythropoietin to its receptor leads to an activation of the phosphatidylinositol 3-kinase/Akt pathway and NF-kB pathway via phosphorylation resulting in an up regulation of antiapoptotic proteins including XIAP and C-IAP2, subsequently blocking the activation of specific cell-death proteases leading to apoptosis [34]. Erythropoietin mediates its neuroprotective properties via cross-talk between JAK2 and the nuclear factor-kB (NF-kB) [35]. More recently, it was shown for neurons in the central nervous system that erythropoietin mediates regeneration via JAK2 and the phosphatidylinositol 3-kinase/Akt pathway [36].

In summary, our study clearly demonstrated the high neuroprotective efficacy of rhEPO in the peripheral nervous system with respect to neurophysiological functions and for the morphological integrity. Additionally the somatic hypersensitivity showed tendency to improvement. The effect of rhEPO is not mediated by attenuated DNA adduct formation or accelerated repair. The mitochondrial protection and significantly increased frequency of the mitochondria might contribute to the neuroprotective property of rhEPO. Further clinical trials of erythropoietin in patients with peripheral neuropathy seem to be warranted in the future.

## Methods

### Animals

Male C57Bl/6/J (group A-D, n_A-D _= 40) mice from the breeding stock of the Institute for Cell Biology were used throughout all experiments. Mice were housed under specific pathogen free (SPF) conditions with 12 h/12 h dark-light cycle, fed with 1314 fortified standard diet (Altromin, Germany) and water ad libitum. Mice were aged 20 weeks at the time of behavioral testing and neurophysiological examination. All experiments were approved by the state animal welfare board (G-033/05Z).

### Study design

Mice were randomly divided into four groups (group A-D, n_A-D _= 40). All mice were treated for eight weeks. Group A was treated with cisplatin (Platinex^®^, Bristol, Munich, Germany) 2 mg/kg i.p. once per week and group B was additionally treated with rhEPO (Neorecormon^®^, Roche, Basel, Switzerland) 40 μg/kg s.c. three times per week [37]. To prevent renal damage all mice were injected with 500 μl saline i.p. after cisplatin injection. Group C were treated with rhEPO 40 μg/kg s.c. three times per week and group D were treated with saline 500 μl i.p. once per week. In week eight all mice were tested for their renal function prior to behavioural testing and nerve conduction velocity studies. Thereafter, the mice were sacrificed, blood was taken from the heart to quantify serum creatinine and the sciatic nerves were removed for histological analysis.

### Electrophysiological examination of motor and sensory nerve function

All electrophysiological examinations were carried out under general anesthesia with Thiobutabarbital sodium salt hydrate (Inactin^®^, Sigma; i.p. 50 μg/kg b.w.). Sensory- and motor nerve conduction velocities were calculated using recordings of H- and M- responses following stimulation of sciatic/tibial nerves as described previously [38].

The compound muscle action potentials (CMAPs) elicited by orthodromic conduction (M-response) and by the monosynaptic reflex arc (H-response), were recorded from the fourth interosseous muscle of the hind paw with a pair of micro needle recording electrodes. After amplification (INH 4-channel differential amplifier, Science Products, Hofheim, Germany) the evoked action potentials were sampled and analyzed by means of a CED micro1401 system with the "Signal for Windows Version 2.05" software (Cambridge Electronic Design, Cambridge, UK)

Peak-to-peak amplitudes of M- and H-responses were measured. The response latencies (ms) were defined as the interval between the stimulus artifact and the beginning of the M- or H-response. The distance between the two stimulation points was measured over the skin with the hip and knee in a flexed position. Motor (MNCV) and H-reflex-related sensory nerve conduction velocity (SNCV) were calculated as follows:

MNCV [m/s] = distance (sciatic notch-ankle)/latency (M-response sciatic notch - M-response ankle);

SNCV [m/s] = distance (sciatic notch-ankle)/latency (H-response sciatic notch - H-response ankle). All groups were blinded to the examiner.

### Mechanical sensitivity

To assess changes in mechanical nociceptive thresholds, mice were placed on a plastic mesh floor covered by transparent plexiglas cages. For testing, mice were allowed to acclimatize for 30 minutes. Successively greater diameter von Frey nylon monofilaments (Stoelting, USA) were applied to the hind paw. The mechanical sensitivity was assessed using the up and down method [39]. The 50% probability withdrawal threshold (force of the von Frey hair to which an animal reacts to 50% of the presentations) was recorded. To reduce the amount of experimental animals a fourth group treated with rhEPO was abdicated since rhEPO did not have any effect on the nerve conduction velocity studies. All groups were blinded to the examiner.

### Electron microscopic examination of sciatic nerves

Sciatic nerves were fixed in 2.5% glutaraldehyde in phosphate buffer immediately after removal. After washing in phosphate buffer, tissue samples were osmicated for 1 h with 1%OsO_4 _in phosphate buffer, dehydrated in a graded series of alcohols, and embedded in Epon 812 (Shell Chemical of Houston, Texas, USA). Electron microscopic examination was performed using a Zeiss EM 902. For statistical analysis of the mitochondria a total of 150 axons in 12 nerves each (3 × group A, 3 × group B, 3 × group C, 3× group D) were examined. All sections were blinded to the examiner.

### Quantification of Pt-DNA adducts by the Immuno-Cytological Assay (ICA)

Immunofluorescence staining and quantification of specific platinum adducts in the nuclear DNA was performed essentially as described previously with some modifications [25]. Briefly, 7 μm thick DRG tissue sections were fixed in methanol (12 h; -20°C) and rehydrated in PBS (10 min; 25°C). After denaturation by alkali treatment (60% 70 mM NaOH/140 mM NaCl, 40% methanol; 5 min, 0°C) cytoplasmic and nuclear proteins were digested by incubation with pepsin (Sigma, 300 μg/ml in 20 mM HCl; 10 min, 37°C). After blocking (1% casein/PBS; 30 min; 25°C) slides were immunostained for DNA adducts with an anti-(Pt-GG) monoclonal antibody (MAB R-C18; 0.2 μg/ml) and with Cy3-rabbit anti-(rat-Ig) (Dianova, Hamburg, Grermany). The DNA was counterstained with DAPI (1 μg/ml PBS).

The quantification of fluorescence signals was performed by a microscope-coupled digital image analysis system (Zeiss Axioplan and ACAS 6.0 Image Analysis System, Ahrens Electronics; Bargterheide, Germany). Adduct levels in the nuclear DNA of individual cells were calculated by normalizing antibody-derived fluorescence signals to the corresponding DNA content of the same cell and are expressed as arbitrary fluorescence units (AFU). Cryosections from 5 mice per group were analyzed. Mean values ± SEM of > 400 cells were calculated.

### Renal function test

To test renal function, mice were placed in metabolic cages for 24 hours and urine was collected in a cooled (4°C) container to minimize evaporation. Thereafter blood was taken from the heart. Creatinine levels were determined in urine and serum samples using the Jaffé method (ADVIA 1650 Chemistry System, Bayer Health Care, Fernwald, Germany). Creatinine Clearance as a marker of glomerular filtration was calculated as urine creatinine × urine volume/serum creatinine × 1440 min. All four groups were blinded to the examiner.

### Statistical analysis

One factorial ANOVA (between subject factor GROUP: A vs. B vs. C vs. D) was performed to compare mean amplitudes of H- and M responses and mean sensory and motor conduction velocities in the groups A-D. Post-hoc comparisons were performed with multiple T-tests with Bonferroni corrections. The level of significance was 0.05.

## Abbreviations

DRG: dorsal root ganglia; EPO: erythropoietin; EpoR: erythropoietin receptor; rhEPO: recombinant human erythropoietin; HMGB1: high-mobility group 1 protein; mtDNA: mitochondrial DNA; NER: nucleotide excision repair; Pt-GG: platinum guanine-guanine intrastrand cross-link

## Authors' contributions

MSY conceived the study, participated in design and coordination, performed the electrophysiological studies and drafted the manuscript. ZK carried out the study design and revised the manuscript. MO carried out the statistics. MS performed the mechanical sensitivity. BL made substantial contributions to conception of the study. AD carried out the cryosections and the Immuno-Cytological Assays. AK performed renal function tests. RE carried out the micro morphological analysis. VL made contributions to conception of the study. HCD participated in the design of the study and revised the manuscript. JT conceived the study, participated in its design and coordination and revised the manuscript critically and gave the final approval to be published. All authors read and approved the final manuscript.
